# Medical and Dental Departments, Central University of Kentucky

**Published:** 1889-09

**Authors:** 


### Medical and Dental Departments, Central University of Kentucky.

The commencement exercises of the Medical and Dental Departments of the Central University of Kentucky (Louisville College of Dentistry and Hospital College of Medicine), took place at Louisville, Ky., June 18. The classes in both departments were the largest ever graduated, and there were more than twice as many students as last year, while the graduates, eighty in all, were more than three times as many.

The honor men were Noble R. Townsend, of Arkansas; William S. Noblette, of Indiana, and Charles T. Duncan, of Kentucky. These three had the same average, and decided among themselves who should receive the prizes to be awarded. Dr. Townsend took the curator's medal, Dr. Noblette the faculty medal, and Dr. Duncan the position of resident physician to the city hospital, which is regarded as the best of the three prizes. The dental faculty medal was awarded to Dr. S. T. Butler.

